# Effect of 1,3-1,6 β-Glucan on Natural and Experimental Deformed Wing Virus Infection in Newly Emerged Honeybees (*Apis mellifera ligustica*)

**DOI:** 10.1371/journal.pone.0166297

**Published:** 2016-11-09

**Authors:** Maurizio Mazzei, Baldassare Fronte, Simona Sagona, Maria Luisa Carrozza, Mario Forzan, Federica Pizzurro, Carlo Bibbiani, Vincenzo Miragliotta, Francesca Abramo, Francesca Millanta, Marco Bagliacca, Alessandro Poli, Antonio Felicioli

**Affiliations:** 1 Department of Veterinary Science, University of Pisa, Pisa, Italy; 2 Scuola Normale Superiore, Pisa, Italy; University of Otago, NEW ZEALAND

## Abstract

The Western Honeybee is a key pollinator for natural as well as agricultural ecosystems. In the last decade massive honeybee colony losses have been observed worldwide, the result of a complex syndrome triggered by multiple stress factors, with the RNA virus Deformed Wing Virus (DWV) and the mite *Varroa destructor* playing crucial roles. The mite supports replication of DWV to high titers, which exert an immunosuppressive action and correlate with the onset of the disease.

The aim of this study was to investigate the effect of 1,3–1,6 β-glucan, a natural innate immune system modulator, on honeybee response to low-titer natural and high-titer experimental DWV infection. As the effects exerted by ß-glucans can be remarkably different, depending on the target organism and the dose administered, two parallel experiments were performed, where 1,3–1,6 ß-glucan at a concentration of 0.5% and 2% respectively, was added to the diet of three cohorts of newly emerged honeybees, which were sampled from a *Varroa*-free apiary and harboured a low endogenous DWV viral titer. Each cohort was subjected to one of the following experimental treatments: no injection, injection of a high-copy number DWV suspension into the haemocel (experimental DWV infection) or injection of PBS into the haemocoel (physical injury). Control bees fed a ß-glucan-free diet were subjected to the same treatments. Viral load, survival rate, haemocyte populations and phenoloxidase activity of each experimental group were measured and compared. The results indicated that oral administration of 0.5% ß-glucan to naturally infected honeybees was associated with a significantly decrease of the number of infected bees and viral load they carried, and with a significant increase of the survival rate, suggesting that this natural immune modulator molecule might contribute to increase honeybee resistance to viral infection.

## Introduction

The Western honeybee is the best known and widely managed pollinator species, whose services to agriculture have been estimated at $ 200 billion per year worldwide [1].

Honeybees have recently suffered extensive losses worldwide [2]. This phenomenon is not new, as it has repeatedly been reported in the Northern hemisphere since the second half of the nineteenth century [3]. The intensive growth of the bee-keeping industry and the huge losses observed in the last two decades prompted the scientific community to undertake intensive research to investigate the causes of these recurrent outbreaks. Exposure to pesticides, malnutrition and pathogens, including bacteria, fungi, mites, and RNA viruses have been in turn identified as the main threat to bee health [4–6]. However, there is now a general consensus that colony mortality is the product of multiple factors, acting singly or synergistically [7, 8]. Among them viruses play a prominent role, which was long not appreciated, as most of them do not induce apparent disease [9]. However, in recent years evidence has emerged that the association of these viruses with the mite *Varroa destructor* is a contributing factor in the collapse of honeybee colonies worldwide [10].

Deformed Wing Virus (DWV) is one of the main viruses associated with honeybee colony losses [11, 12]. DWV can be transmitted vertically and persist in the bee colony as covert infection. Overt infections, characterized by deformities and premature death, leading ultimately to the collapse of the colony, are usually observed in apiaries concomitantly infested by *Varroa* [13, 14]. The mite supports replication of DWV to high titers, which exert an immunosuppressive action [14], and correlate with the onset of clinical signs [15], consisting of crumpled and/or vestigial wings and a bloated abdomen [11]. The symptomatic bees die soon after emergence. Asymptomatic bees can also be heavily infected, though with lower viral titers. During the first stage of the infection DWV is only detected in the abdomen, at later stages presence of an actively replicating virus is readily detectable in the head [16–18]. Although honeybees, like all insects, lack an acquired immune system, they have a well developed innate immune system, which can generate a wide array of non-specific humoral and cellular responses triggered by the recognition of pathogen-associated molecular patterns (PAMPs) such as bacterial peptidoglycans, fungal ß-glucans and viral dsRNA, by host receptors (PRRs—Pathogen Recognition Receptors) [19]. The humoral response includes antimicrobial peptides (AMPs), reactive intermediates of oxigen or nitrogen and the enzymatic cascades that regulate coagulation or melanization of haemolymph [20, 21]. The cellular immunity is mediated by haemocytes, whose response includes phagocytosis, nodulation and encapsulation [22, 23]. Nodulation and encapsulation depend on melanization (synthesis and deposition of melanin around the pathogen) [24, 25]. Activation of melanogenesis in turn depends on several intermediate products and enzymes, the most important being phenoloxidase (PO) [26]. Phenoloxidase is expressed as an inactive zymogen (proPO), converted to active PO by proteases [27]. Activation of proPO, as well as other concomitant immune reaction, are triggered by the presence of immuno modulators of microbial origin, such as 1,3 ß-glucans, lipopolysaccharides, and peptidoglycans [28]. ß-glucans are a heterogeneous group of glucose homopolymers found in fungi, plants, algae and some bacteria, which exert a strong immune-stimulatory activity in a wide variety of vertebrate and invertebrate species [29]. To our knowledge, the effects of ß-glucans on honeybee resistance to viral infections have not been analysed so far. As they are non-toxic, non-polluting, highly stable, and biodegradable, they might possibly represent a safer alternative to synthetic pharmacological immune modulators [29–31]. Investigation of the immune response to ß-glucans in insects, particularly in Drosophila, has highlighted the antimicrobial role of Toll and Imd signaling pathways [32]. Antiviral defense in insects is achieved mainly via RNA interference (RNAi); however recent data suggest that Toll and Imd pathways also contribute to defense against viral pathogens [33, 34]. Insects may not completely rely on RNAi to fight against RNA viruses because these viruses have developed ways of suppressing RNAi responses at different stages of RNAi pathway, as observed in honeybees infected with DWV [35]. These findings prompted us to investigate whether the administration of 1,3–1,6 ß-glucan would modify honeybee resistance to endogenous and/or experimental viral infection. The response triggered by ß-glucans can be remarkably different depending on the target organism and the dose administered [29, 36]. To this purpose two parallel experiments were performed, in which 1,3–1,6 ß-glucan, at a concentration of 0.5 and 2% respectively, was added to the diet of three cohorts of newly emerged honeybees, which were sampled from a *Varroa*-free apiary and harboured a low endogenous DWV viral titer. Each cohort was subjected to one of the following experimental treatments: no injection, injection of a high-copy number DWV suspension into the haemocel (experimental DWV infection) and PBS injection into the haemocoel (physical injury). Control bees fed a ß-glucan-free diet were subjected to the same treatments. Viral load, survival rate, haemocyte populations and phenoloxidase activity of honeybees fed in presence or absence of β-glucan were measured and compared.

## Materials and Methods

### Honeybees and experimental design

**Fig 1:**
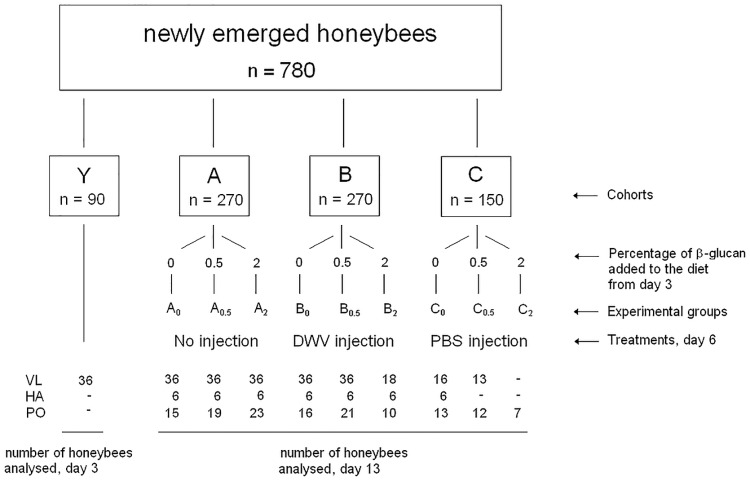
Experimental design diagram. The number of individual honeybees constituting each cohort and group, and the number of honeybees analysed for each group are shown. (VL: Viral load; HA: Haemocyte analysis; PO: Phenoloxidase activity).

In May 2015, newly emerged honeybees (*Apis mellifera ligustica* L.) were transferred from a *Varroa*-free hive located in Gorgona Island (43°26′N 9°54′E) [37] to the Department of Veterinary Science of Pisa University (Italy). Samplings were performed to generate three replicates of the experiment, with a total of 780 newly emerged honeybees used in the study. Bees were divided in four experimental cohorts: ‘Y’ (n = 90), ‘A’ (n = 270), ‘B’ (n = 270) and ‘C’ (n = 150). Cohort A was not subjected to any injection. Cohorts B was injected with a viral suspension obtained from DWV infected, symptomatic bees; cohort C was injected with phosphate-bufferd saline (PBS). Each cohort was further divided in three groups named ‘_0_’, ‘_0.5_’ and ‘_2_’ (Fig 1).

Bees belonging to groups Y, A_0_, B_0_ and C_0_ were fed a commercial sugar solution consisting of 19% glucose, 35% fructose, 12% disaccharides, 6% trisaccharides and 6% polysaccharides (Fruttosweet 45, A.D.E.A.). Groups A_0.5_, B_0.5_, C_0.5_ and A_2_, B_2_, C_2_ were fed from day 3 the same sugar solution containing 0.5% and 2% (w/w) 1,3–1,6 β-glucan, respectively. MacroGard (Biorigin, Brazil) was used as a source of 1,3–1,6 ß-glucans. Twenty five small cages with a maximun of 30 (mean 27.2; median 30; range 10–30) honeybees per cage were kept at 28°C for 13 days, with the exception of the bees belonging to cohort Y, which were sacrificed at day 3 to ascertain their DWV status. Experimental cages were kept at 28°C since in preliminary trials we observed that setting the temperature at 34°C generated a high level of moisture, detrimental to bee survival. Setting the temperature at 28°C was a good compromise to avoid any moisture effect without affecting the well-being of bees. Dead individuals were counted and removed twice per day.

### Preparation of a viral suspension from DWV infected, symptomatic bees

Four DWV-infected honeybees were collected from a Varroa mite infested apiary kept at the Department of Veterinary Science of Pisa University in San Piero a Grado and processed as previously described [18]. Briefly, the bees were deprived of the poison sac, pooled, homogenized with TissueLyser II (Qiagen, Hilden, Germany) for 3 min at 25 MHz in presence of 1 ml of phosphate-buffered saline (PBS) pH7,2 and centrifuged at 14000 rpm for 5 min. The supernatant was sterilized by filtration through a 0.22 μm filter and stored at 4°C in the dark. The suspension was shown by qRT-PCR to contain 5x10^8^ copies of DWV/μl. A dilution containing 4x10^4^ DWV copies per microliter was prepared and used for the injection experiment.

### Injection assay

Bees of cohorts B and C were anaesthetized by CO_2_ inhalation and injected at day 6 into the thorax haemolymph with 2.5 μl of a diluted viral suspension, containing 1x10^5^ DWV copies and with PBS pH7.2, respectively. Bees belonging to cohort A were also anesthetized by CO_2_ inhalation to be exposed to the same experimental condition as those of cohorts B and C.

### Honeybee sample processing

All bees were anesthetized by CO_2_ inhalation before processing. Bees belonging to cohorts Y, A, B and C used for the analysis of the viral load were sacrificed, individual heads and abdomens were dissected immediately, soaked into RNAlater solution (Qiagen) and stored at -80°C until processed. Haemolymph was collected from 42 honeybees belonging to cohort A, B and C. Bees of cohorts A, B and C used for the phenoloxidase assay were stored at -20°C until processed.

### Quantification of DWV viral load

Total RNA extraction and DWV absolute quantification were performed as previously described [18]. Briefly, individual heads and abdomens were separately homogenized using a TissueLyser II (Qiagen), total RNA was extracted with RNeasy mini Kit (Qiagen), eluted in 30 μl RNase-free water and quantified with RiboGreen RNA Quantitation Kit (Invitrogen, Carlsbad, CA, USA). Ten microliters of each RNA were used as template to determine the viral load by RT-qPCR. Samples with RT-qPCR amplification signal were considered positive and those without signal negative. Results were expressed as viral copy number per microgram of RNA.

### Survival analysis

To determine whether oral administration of 1,3–1,6 ß-glucan might affect the bee life span, survival rate was calculated as the percentage of the individuals of each experimental group which survived until day 13.

### Haemocyte analysis

Haemolymph of 42 honeybees belonging to the cohorts A, B and C was analysed. One microliter of haemolymph was collected using a glass capillary introduced in the *sinus dorsalis*, between the third and fourth tergite [38]. The slides were immediately prepared and stained with Diff Quik^®^. Slides with less than 100 cells were considered inadequate for analysis. Haemocyte subtype analysis for each individual honeybee was done by optic microscopy (magnification 40X and 100X) on 10 high power fields. Based on morphology, the haemocytes were classified as prohaemocytes, plasmatocytes, granulocytes and oenocytoids [38, 39].

### Phenoloxidase activity

To determine the phenoloxidase (PO) activity, each head was soaked in 200μl of 50mM PBS pH7.4, 1% Triton X-100 at -20°C for 20 min, homogenized, centrifuged at 4000 rpm at 4°C for 15 min and the supernatant collected. The pellet was resuspended in 200μl of 50mM PBS pH7,4 and centrifuged as above. The supernatants were combined and protein concentration was measured by Qubit fluorimeter (Invitrogen). The enzymatic activity was determined according to Alaux et al. [40] using thirty μg of total protein. Results were expressed as mUE/min/mg of tissue.

### Statistical analysis

Statistical analysis was performed using SAS Institute, 2008 (JMP Statistics and Graphics Guide. SAS Institute Inc., Cary, NC, USA). The estimated survival rate was analysed by Wilcokson rank test using the product-limit (Kaplan-Meier) method for more factors of right-censored data. When factors significantly differed from homogeneous distribution, paired tests were performed on pre-ordered means [41]. PO analysis was performed by the following criteria: data residues obtained by preliminary ANOVA for more factors (treatments, doses and interaction treatment x doses) were tested for normal distribution. Since their distribution differed significantly from the normal distribution, these data were analysed by non parametric Kruskal-Wallis Test. The same analysis was done for viral load and haemocyte counts [42]. When significant differences were found between factors, means were ordered and differences were again tested with non parametric Kruskal-Wallis Test.

## Results

### DWV viral load

**Fig 2:**
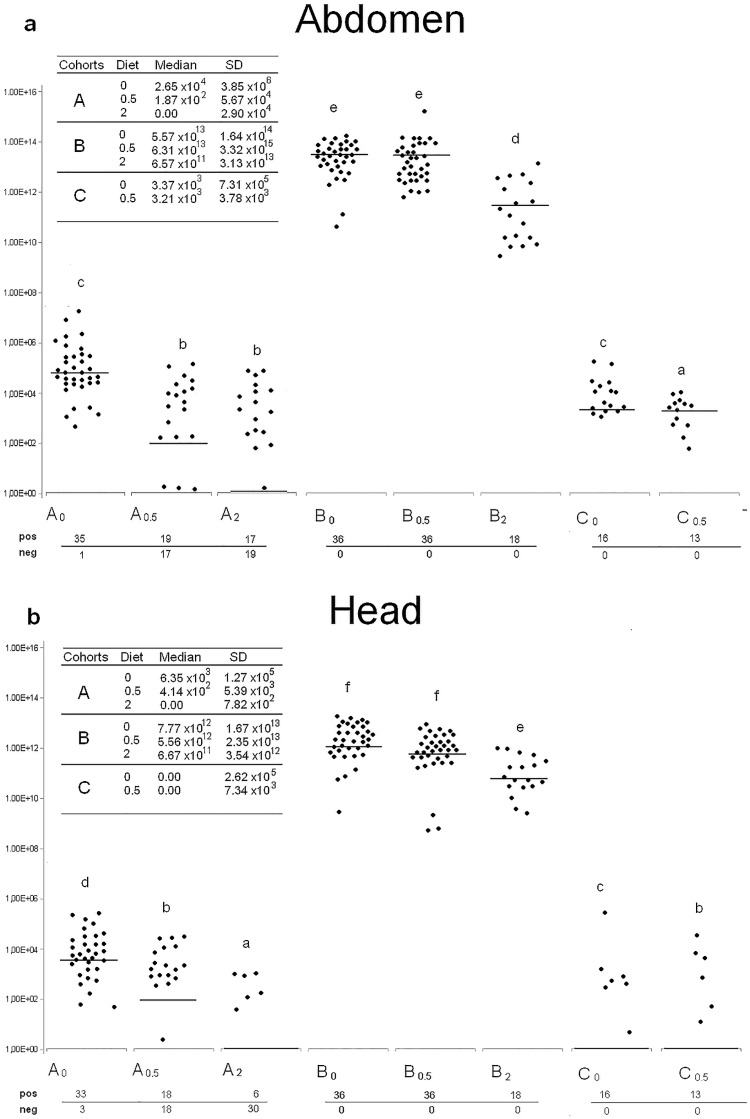
DWV viral load. Viral load values of individual abdomens (a) and heads (b) are shown for cohort A, B and C respectively. Median and standard deviation values are shown in the box. Black dots (●) represent individual DWV viral load values per μg of RNA. Horizontal lines indicate the median. Different letters on top of boxplot indicate statistically significant differences among groups (p<0.01). Number of positive and negative samples within each experimental group are shown.

Abdomens and heads of 36 honeybees belonging to cohort Y were analysed at day 3 by qRT-PCR to ascertain DWV presence and load in the population selected for the study: 20 abdomens and 7 heads harboured the viral genome, with DWV copy numbers below 3 x10^2^ and 5x10^1^respectively. At day 13, the analysis of the viral load in the experimental cohorts (Table 1a) revealed significantly different values in the abdomens as well as in the heads (p<0.01). ß-glucan dosage (Table 1b) significantly affected viral load values in abdomens (p<0.01), whereas viral loads in the heads were only significantly reduced by 2% ß-glucan. With regard to the effect of ß-glucan dosage, group A_0_ abdomens were all positive except one, with viral loads ranging from 10^3^ to 10^7^. Notably, only 19 A_0.5_ and 17 A_2_ abdomens carried the virus, with lower viral loads (p<0.01), not exceeding 1 x10^5^ (Fig 2a). A similar trend was observed for the heads, with 33 positives in A_0_ group, 18 in A_0.5_ and only 6 in A_2_, with viral loads up to 5 x10^5^, 2 x10^4^ and 3 x10^3^ respectively (Fig 2b).

**Table 1:** Viral load analysis.

A	**Cohort A**	**Cohort B**	**Cohort C**
**Abdomen**	1,4 x10^3^ *(2*.*2 x10*^*6*^*)* **a**	3,9 x10^13^ *(2*.*1 x10*^*15*^*)* **b**	5,3 x10^3^ *(5*.*2 x10*^*5*^*)* **c**
**Head**	4,1 x10^2^ *(7*.*7x10*^*4*^*)* **a**	4,8 x10^12^ *(1*.*9 x10*^*13*^*)* **b**	3,1 x10^1^ *(1*.*8 x10*^*5*^*)* **c**
B	**no ß-glucan**	0.5% ß-glucan	**2% ß-glucan**
**Abdomen**	2.7 x10^5^ *(1*.*2 x10*^*14*^*)* **a**	1.7 x10^4^ *(2*.*2 x10*^*15*^*)* **b**	9.2 x10^3^ *(1*.*8 x10*^*13*^*)* **c**
**Head**	3.8 x10^4^ *(1*.*2 x10*^*13*^*)* **a**	4,4 x10^3^ *(1*.*7 x10*^*13*^*)* **a**	0.0 *(2*.*1 x10*^*12*^*)* **b**

(A) Viral load values of abdomens and heads are shown for cohort A, B and C. (B) Viral load values detected in honeybees fed no-ß-glucan, 0.5% and 2% ß-glucan. Median and standard deviation (within brackets) are reported. Different letters indicate statistically significant differences.

Abdomens and heads of all honeybees belonging to cohort B, experimentally infected by injection of 1x 10^5^ viral particles, harboured the virus with most viral loads ranging from 3.5x 10^10^ to 1.4x 10^14^ and from 2.5x 10^9^ to 8.3x 10^13^, respectively. These values were about 10 orders of magnitude higher than those of group A (p<0.01). Abdomen and head viral load values of B_2_ were significantly lower than the other B groups.

Cohort C results were similar to cohort A (Fig 2a and 2b); however, due to the low number of honeybees available for group C_2_, statistical analysis was not performed.

### Survival analysis

**Fig 3:**
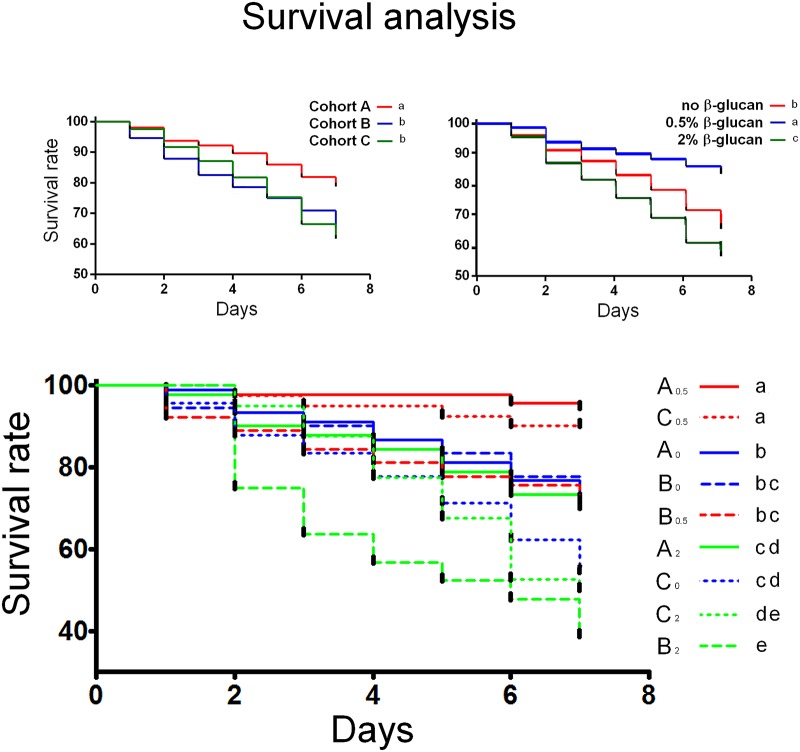
Survival analysis. (a) Survival rate of cohorts A, B and C. (b) ß-glucan dosage effect on survival rate. (c) Survival rate of individual groups; groups are shown on the right side of the figure, according to decreasing survival rate. Colours indicate ß-glucan dosage: blue = no ß-glucan; red = 0.5%; green = 2%. Continuous line = cohort A; dashed line = cohort B; dotted line = cohort C. Different lower case letters indicate statistical differences (p<0.05).

Cohort A had the highest survival rate (p<0.01); no differences were observed between cohorts B and C (Fig 3a). With regard to the effect of ß-glucan dosage the highest and lowest survival rates were associated with 0.5% and 2% ß-glucan, respectively (Fig 3b; p<0.05). Both naturally infected groups fed 0.5% ß-glucan (A_0,5_ and C_0,5_) had the highest survival rate among all groups and cohorts whereas the administration of 0.5% ß-glucan did not increase the survival rate of the experimentally infected bees (B_0,5_) with respect to the groups fed no- ß-glucan. The administration of 2% ß-glucan significantly reduced the survival rate within cohorts A and B, with groups B_2_ and C_2_ showing the lowest survival rate among all groups and cohorts (Fig 3c).

### Haemocyte analysis

**Fig 4:**
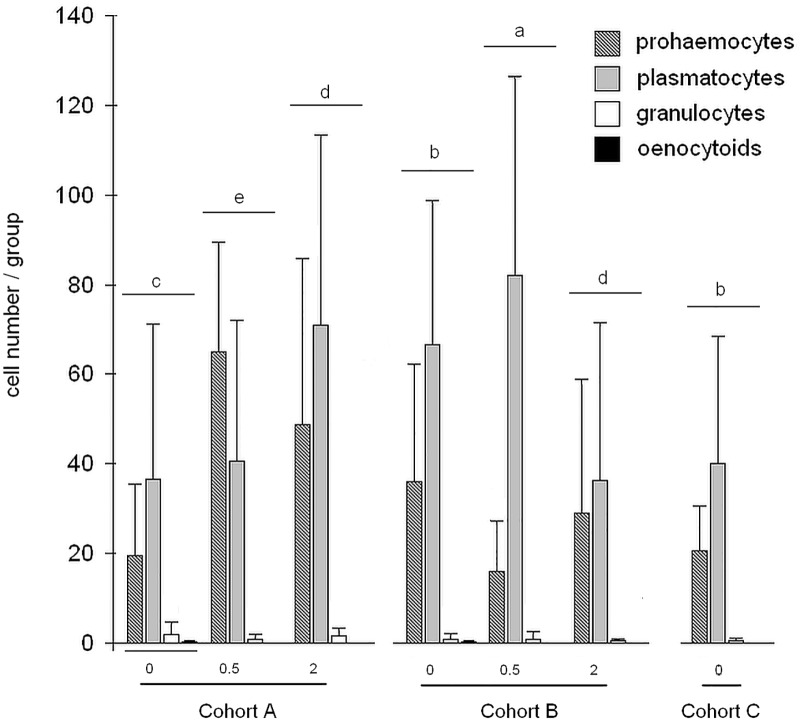
Histogram of cell profile per group. Bar graph indicates number of haemocyte subtypes / groups. Different letters on top of boxplot indicate statistically significant differences among groups (p<0.05).

Four haemocyte types were observed in each group: prohaemocytes, plasmatocytes granulocytes and oenocytoids. The cell profile of cohort A differed significantly from cohorts B and C (p<0.01) Statistical analysis of the effects of ß-glucan dosage indicated significant differences (p<0.01). The interaction between treatments and ß-glucan dosage was statistically significant (p<0.01). The analysis of cellular patterns (Fig 4) indicated significant differences among groups (p<0.05). The investigation of individual cell types, showed that only prohaemocytes differed significantly. Cohort A honeybees, in particular A_0.5_ and A_2_ groups, showed the highest prohaemocyte counts among all cohorts and groups (p<0.05).

### Phenoloxidase activity

The PO activity of cohorts A, B and C did not differ significantly. On the other hand, the ß-glucan dosage affected the PO activity (46.6 ± 22.3; 51.5 ± 27.3; 37.9 ± 14.8 mUE/min/mg in groups fed 0, 0.5 and 2% ß-glucan respectively, p<0.01). The PO activity within each cohort was also affected by ß-glucan dosage (p<0.01). In particular, among all groups analysed, the PO values were comprised between 56.2 ± 40.1 and 36.9 ± 13.4 mUE/min/mg, with both values detected in cohort A (A_0.5_ and A_2_ respectively). The same trend was observed in the other experimental cohorts where honeybees fed 2% ß-glucan showed lower PO activity than those fed 0.5% ß-glucan.

## Discussion

The pathogen-associated decline of pollinator species is a serious threat for agricultural as well as natural ecosystems that may have profound economic and environmental consequences worldwide. The aim of this work was to investigate whether the oral administration of 1,3–1,6 ß-glucan, a natural immune-modulator, could contribute to the response of honeybees to low-titer natural and high-titer experimental DWV infection. Viral load, survival rate, haemocyte types and phenoloxidase activity were evaluated and compared between 1,3–1,6 ß-glucan fed and unfed honeybees. As the experimental infection involved injection of virus into the haemolymph, a cohort of honeybees was injected with saline solution only, in order to ascertain whether the injection (physical injury) itself might affect some of the parameters under study. The immune status of the honeybees depends on age [43] and behavioural role, as demonstrated by Bull et al. [44] who compared survival rate and expression of immune genes in forager and house honeybees experimentally infected with a pathogenic fungus. Forager bees survived significantly longer than the younger house honeybees, their greater resistance being associated with the developmentally regulated increased expression of genes that control the production of antimicrobial proteins [44]. All honeybees used in this study were of the same age, as we collected newly emerged bees from brood frames removed from an apiary maintained on the Gorgona Island, where the disappearance of the *Varroa* mite recorded during the last three years was associated with low DWV viral loads [37]. Consistent with these findings, at day 3, before ß-glucan was administered to cohorts A, B and C, twenty out of thirty six honeybees belonging to cohort Y exhibited barely detectable numbers of viral genome in the abdomen and only seven in the head. When, at day 13, we analysed the number of DWV-positive A_0_ abdomens and heads and the respective viral loads, it was clear that the endogenous infection had progressed during the course of the experiment, since the number of DWV-positive abdomens and heads as well as the viral load values were significantly higher than the corresponding values of cohort Y. Interestingly, day 13 analysis of ß-glucan-fed A_0.5_ and A_2_ groups clearly revealed that the numbers of DWV-positive abdomens and heads and the respective viral loads were significantly lower than the A_0_ corresponding values. Although we could not analyse each individual bee during the course of the experiment, we can hypothesize that ß-glucan restrained the viral replication in bees negative to the RT-qPCR assay as well as in bees positive at day 3. As all bees belonging to cohort B were injected with a high-copy-number viral suspension, they were clearly all positive and therefore only the effect of ß-glucan on the viral load was analysed, revealing a trend similar to the one observed in cohort A, although only group B_2_ significantly differed from B_0_ and B_0.5_. All cohort B viral load values were approximately 10 orders of magnitude higher than those of cohort Y and 7 orders of magnitude higher than those of cohort A. All heads were positive to DWV indicating that the injected DWV suspension contained productive viral particles. The number of viral particles injected in the honeybees most likely exceeded the ability of β-glucan to effectively restrain the viral replication. We cannot exclude the possibility that the suspension used for the injection might contain other bee viruses considering that most of them are asymptomatic. However, it may be assumed that the immune response enhancement triggered by the ß-glucan stimulation might not be restricted to DWV, contributing to the response to other viral bee pathogens as well. Cohort C results were similar to cohort A, however only C_0_ and C_0.5_ were analysed, as the number of C_2_ honeybees available for viral load analysis was too low to allow a statistical analysis to be performed.

The survival rate of cohort A was significantly different from DWV and PBS injected cohorts B and C. It has been reported that the effects exerted by ß-glucans can differ dramatically, depending on the target organism and the dose administered [29]. Consistent with these observations, 0.5% ß-glucan significantly increased the survival rate with respect to a ß-glucan-free diet, while administration of 2% ß-glucan was associated with a significantly lower survival. With regard to the analysis of individual groups, we observed that 0.5% ß-glucan was associated with a significantly higher survival of naturally infected _A0.5_ and C_0.5_ groups. The survival rate of A_2_ group was the lowest within the cohort while C_2_ group survival was unaltered with respect to C_0_. Cohort B survival response to ß-glucan was remarkably different, in that 0.5% ß-glucan did not affect the survival rate, while administration of 2% ß-glucan, even though it restrained the viral replication, appeared to be detrimental to the survival rate of B_2_ honeybees, the lowest one among all cohorts and groups. Interestingly, this result did not seem to be caused exclusively by the extremely high viral load harboured by all B honeybees, as groups B_0_ and B_0.5_ had the same survival rate as groups A_0_ and A_2_ whose viral loads were orders of magnitude lower. It can be speculated that the immune pathways’ activation triggered by 2% ß-glucan in presence of high viral load might adversely affect the host, as observed by Fronte et al. (personal communication). In agreement with previous reports [39, 45], plasmatocytes were the most represented haemocyte subtype in our study population, except in group A_0.5_. This finding may support what previously reported by Sabcaliu et al. [38] that described an increase of plasmatocytes in haemolymph of honeybees under stressful conditions. Prohaemocytes were the most represented haemocyte subtype in group A_0.5_ suggesting that 0.5% ß-glucan may exert an immunostimulatory effect in naturally infected honeybees. Szymas and Jedruszuk [46] described a granulocyte decrease in bees fed a pollen substitute containing ß-glucan and this may explain the low percentage of granulocytes in our study. No significant variation in the haemocyte subpopulations was found in ß-glucan-fed cohort B bees.

The PO activation pathways are complex and affected by many factors [28, 47]. It is noteworthy that the highest PO activity was observed in the A_0,5_ group, which also had the highest survival rate, one of the lowest numbers of DWV-infected bees, the lowest viral load values and the highest number of prohaemocytes. On the contrary, the lowest PO activity was observed in B_2_ group which had the lowest survival rate and one of the highest viral load values. Although we did not find any interaction between DWV and ß-glucan administration in relation to the innate immune response (PO activity) the overall results indicate an effect of those on honeybee survival rate.

## Conclusions

In conclusion our findings suggest that the oral administration of 0.5% ß-glucan to newly emerged honeybees harbouring a low-titer natural DWV infection might contribute to increase honeybee resistance, by restraining viral replication, increasing the number of prohaemocytes and the phenoloxidase activity, ultimately prolonging the honeybee lifespan.
